# Metabolic-Hydroxy and Carboxy Functionalization of Alkyl Moieties in Drug Molecules: Prediction of Structure Influence and Pharmacologic Activity

**DOI:** 10.3390/molecules25081937

**Published:** 2020-04-22

**Authors:** Babiker M. El-Haj, Samrein B.M. Ahmed

**Affiliations:** 1 Department of Pharmaceutical Sciences, College of Pharmacy and Health Sciences, University of Science and Technology of Fujairah, Fufairah 00971, UAE; 2 College of Medicine, Sharjah Institute for Medical Research, University of Sharjah, Sharjah 00971, UAE; samahmed@sharjah.ac.ae

**Keywords:** alkyl moieties, hydroxy functionalization, carboxy functionalization, prodrug metabolic activation, primary and auxiliary pharmacophores, auxophores

## Abstract

Alkyl moieties—open chain or cyclic, linear, or branched—are common in drug molecules. The hydrophobicity of alkyl moieties in drug molecules is modified by metabolic hydroxy functionalization via free-radical intermediates to give primary, secondary, or tertiary alcohols depending on the class of the substrate carbon. The hydroxymethyl groups resulting from the functionalization of methyl groups are mostly oxidized further to carboxyl groups to give carboxy metabolites. As observed from the surveyed cases in this review, hydroxy functionalization leads to loss, attenuation, or retention of pharmacologic activity with respect to the parent drug. On the other hand, carboxy functionalization leads to a loss of activity with the exception of only a few cases in which activity is retained. The exceptions are those groups in which the carboxy functionalization occurs at a position distant from a well-defined primary pharmacophore. Some hydroxy metabolites, which are equiactive with their parent drugs, have been developed into ester prodrugs while carboxy metabolites, which are equiactive to their parent drugs, have been developed into drugs as per se. In this review, we present and discuss the above state of affairs for a variety of drug classes, using selected drug members to show the effect on pharmacologic activity as well as dependence of the metabolic change on drug molecular structure. The review provides a basis for informed predictions of (i) structural features required for metabolic hydroxy and carboxy functionalization of alkyl moieties in existing or planned small drug molecules, and (ii) pharmacologic activity of the metabolites resulting from hydroxy and/or carboxy functionalization of alkyl moieties.

## 1. Introduction

Nonpolar alkyl moieties are frequently incorporated into drug molecules to serve pharmacodynamic and/or pharmacokinetic purposes. Being lipophilic, alkyl moieties are metabolized in phase I via hydroxy functionalization to alcohols—a process which is, in some cases, followed by carboxy functionalization. Usually, the carboxyl and sterically unhindered hydroxyl groups in the resulting metabolites are conjugated in phase II by glucuronic acid. The chemical forms and metabolic products of the alkyl moieties surveyed in this review are summarized in Table 1.

Generally, alkyls are found in drug molecules in their capacity as functional groups or as frameworks for (or carriers of) hydrophilic or other hydrophobic functional groups. Usually, internal linear alkyls assume the role of frameworks, while ω methyls of terminal linear or branched alkyls assume primary or auxiliary pharmacophoric roles by interacting with biological targets through van der Waals binding. In addition, internal linear alkyls may be used as spacers between functional groups for different purposes—mainly to extend the chain for one of the functional groups to reach a binding site. Further, branching of alkyl chains results in compactness: this feature will cause less disruption of the hydrogen-bonding network of water. Consequently, the lipophilicity of the drug containing the branched alkyl group will decrease, and if the drug’s mechanism of action is related to its lipophilicity, then a significant alteration in the biologic effect will ensue [1].

**Figure 1:**
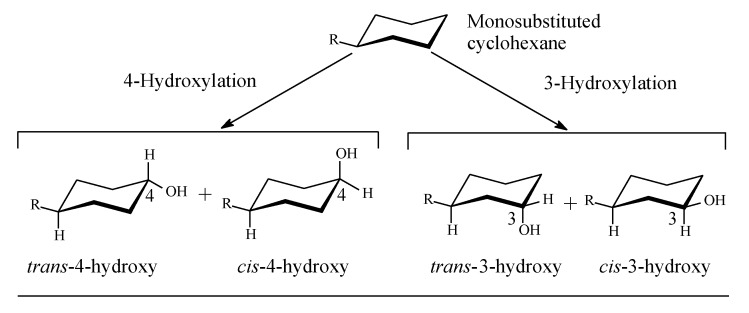
Stereoselective metabolic oxidation of monosubstituted cyclohexyl moiety.

Cycloalkyl groups encountered in drug molecules mostly extend from cyclopropyl to cyclohexyl. In drug design, cycloalkyl groups are substituted for open-chain alkyl groups to (i) better fill a hydrophobic pocket in a receptor, and (ii) introduce rigidity and limit the number of conformations a drug molecule may adopt. Both effects contribute to attaining more drug affinity and selectivity of the drug containing such groups [2,3,4]. In monosubstituted cyclohexyl groups—the most common in drug molecules—metabolic hydroxylation is stereoselective, favoring the *trans* isomer for its higher stability with respect to the *cis* isomer (Figure 1) [5].

### Mechanism of Metabolic Oxidation of Alkyl Moieties

**Figure 2:**
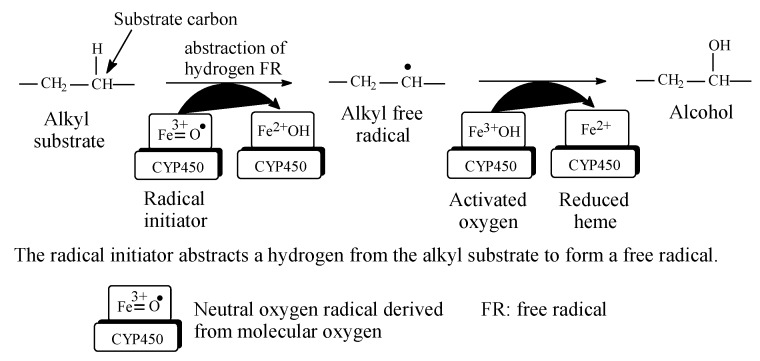
Free-radical metabolic alkyl hydroxylation (adapted from [3,4]).

Metabolic hydroxylation of alkyl groups is catalyzed by a family of monooxygenase enzymes, known as the “cytochrome P450” family, that contain heme redox centers. The heme group is characterized by an iron atom coordinated to the nitrogen atoms of four linked pyrrole rings. The mechanism of metabolic hydroxylation involves free-radical formation at the substrate carbon in the alkyl moiety, as illustrated in Figure 2 [6,7,8,9,10]. In drug molecules containing more than one class of carbon atoms, the priority of metabolic hydroxylation is dictated by the stability of the intermediate free radicals; however, anomalies may occur due to prevailing electronic or steric effects in the molecule. Due to electronic effects, the stability of alkyl free radicals follows this sequence: benzyl, allyl > 3° > 2° > 1° > methyl [11]. The different classes of alkyl carbons are shown in Figure 1.

## 2. Data on Selected Groups of Drugs Containing Alkyl Moieties

The selection of drug candidates for metabolic alkyl-moiety hydroxylation and carboxyl functionalization was based on the presence of the groups in Table 1.

### 2.1. NSAIDS

The chemical classification of NSAIDS is given in the first part of this review series [12]. The two NSAIDS considered in this section, ibuprofen and tolmetin are of the arylalkanoic acid class.

#### 2.1.1. Ibuprofen

**Figure 3:**
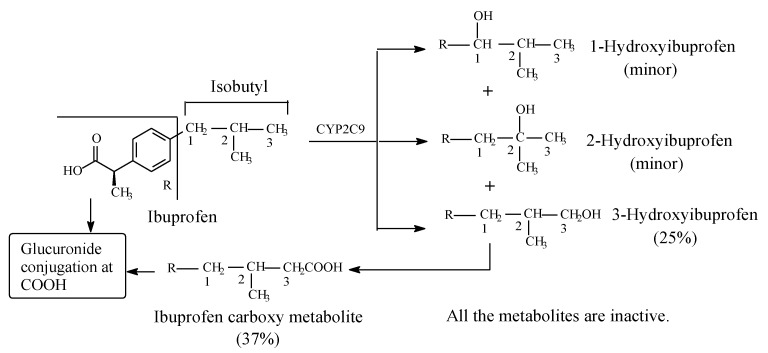
Metabolism of ibuprofen.

**Figure 4:**
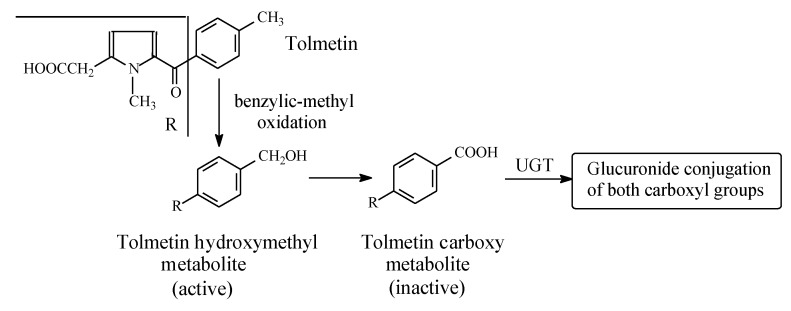
Metabolism of tolmetin.

Ibuprofen (Figure 3) is an arylpropionic acid NSAID used in the management of arthritis as well as for its analgesic and antipyretic properties. It acts as an NSAID by inhibiting COX and consequently PGE_2_, which is implicated in the inflammation process [13]. Ibuprofen is a chiral drug existing in two enantiomeric forms: *S*-(+) and *R*-(−). The NSAID activity of ibuprofen has been reported to reside in the *S*-(+)-enantiomer [14,15,16], which is now marketed in a number of countries as dexibuprofen; however, in most countries, the drug is used as the racemate. The possible reason why large pharmaceutical companies tend to market racemic equivalent ibuprofen is that the *levo* enantiomer is metabolically converted in vivo to the dextro enantiomer [17]. The isobutyl group in ibuprofen contains three classes of carbon: two primary (C3, C3), one tertiary (C2), and one benzylic (C1) (Figure 3). As depicted in Figure 4, phase I metabolic hydroxylation occurs at the three carbons to varying extents [17,18,19,20,21,22]. 3-Hydroxyibuprofen is further oxidized via the aldehyde intermediate to the carboxylic acid metabolite. The benzylic-carbon oxidation results in the formation of a chiral secondary alcohol (1-hydroxyibuprofen). Both the intrinsic and the metabolically generated carboxyl groups are further metabolized in phase II to glucuronide conjugates. All the metabolites of ibuprofen are devoid of pharmacological activity [20,23].

#### 2.1.2. Tolmetin

Tolmetin (Figure 4) is a pyrrole acetic acid NSAID. It is metabolized by the hydroxylation of the benzylic methyl group to the active hydroxymethyl derivative, which is further oxidized to the inactive 5-*p*-carboxybenzoyl-1-methylpyrrole-2-acetic acid in rat, monkey, and human [24,25]. Both the intrinsic and metabolically produced carboxyl groups are further metabolized in phase II to the inactive glucuronide conjugates. The retention of COX-inhibiting activity by the hydroxymethyl metabolite may indicate an auxiliary pharmacophoric role of the benzylic methyl group, since the relatively large phenyl group is responsible for the primary pharmacophoric role.

### 2.2. Sulfonylurea Oral Antidiabetics

**Figure 5:**
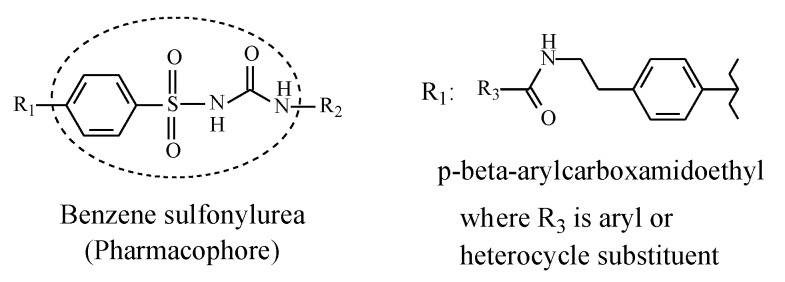
General structure of sulfonylurea antidiabetics depicting the pharmacophore.

Sulfonylurea oral antidiabetics have the general structure shown in Figure 5, with the framed moiety representing the pharmacophore.

In the first-generation sulfonylureas in Figure 5, R_1_ is a small lipophilic group such as methyl or chloro, while R_2_ is a lipophilic alkyl or cycloalkyl group, mostly cyclohexyl. In the second-generation sulfonylureas, the alkyl and cycloalkyl substituents at R_2_ are mostly maintained while the substituent at R_1_ is a large *p*-(β-arylcarboxyamidoethyl) group (Figure 5). This latter group enhances antidiabetic activity through strong binding affinity to the ATP K^+^ channel [26]. On the other hand, according to Foye (2020) [26], the small lipophilic groups at R_1_ in the first-generation sulfonylureas have little influence over activity. Hence, they may have been included to play auxiliary pharmacophoric or auxophoric roles. Nevertheless, only methyl groups at R_1_ are within the scope of this review.

The first-generation sulfonylurea oral antidiabetics surveyed in this review include acetohexamide, tolbutamide, chlorpropamide, and tolazamide, while the second-generation members include glyburide (glibenclamide), glimepiride, and glipizide [27].

Mechanistically, the sulfonylurea antidiabetics act by binding to the specific receptor for sulfonylureas on β-pancreatic cells, blocking the inflow of potassium (K^+^) through the ATP-dependent channel. The flow of K^+^ within the β-cell goes to zero; the cell membrane becomes depolarized, thus removing the electric screen, which prevents the diffusion of calcium into the cytosol. The increased flow of calcium into β-cells causes contraction in the filaments of actomyosin responsible for the exocytosis of insulin, which is therefore promptly secreted in large amounts [28].

#### 2.2.1. Acetohexamide

**Figure 6:**
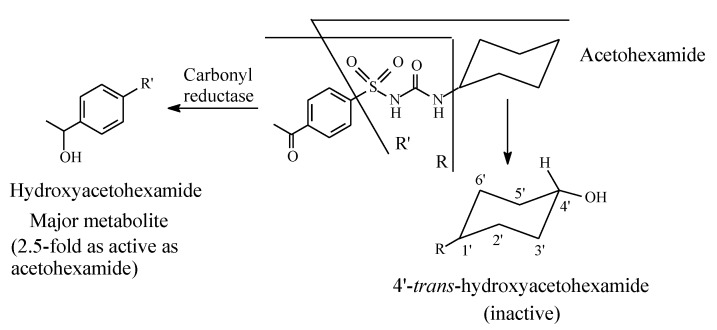
Metabolic pathways of acetohexamide.

Acetohexamide (Figure 6) is metabolized by (i) reduction of the carbonyl group to give a hydroxy metabolite that is 2.5 times as active as the parent drug, as well as (ii) stereoselective oxidation of the cyclohexyl ring to *trans*-4′-hydroxyacetohexamide, which is inactive as an oral antidiabetic [29,30].

#### 2.2.2. Tolbutamide

**Figure 7:**
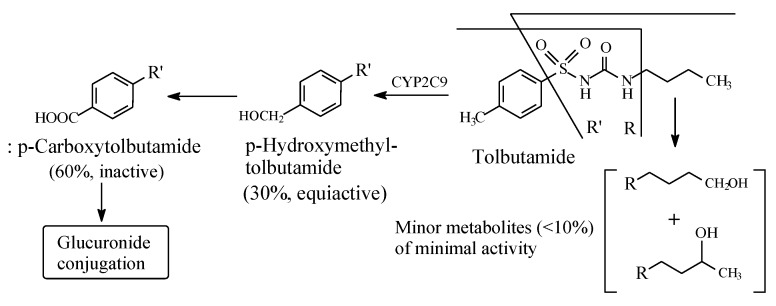
Metabolic pathways of tolbutamide.

**Figure 8:**
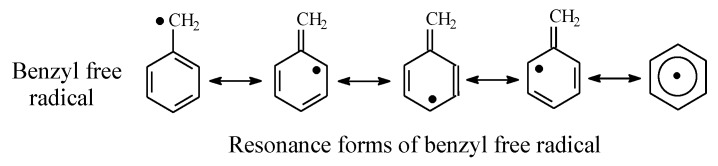
Resonance stabilization of the benzylic free radical.

Tolbutamide (Figure 7) is primarily metabolized by benzylic methyl oxidation to the primary alcoholic hydroxymethyl group, which is further oxidized to the carboxyl group (Figure 7) [31,32]. While the hydroxymethyl metabolite is equiactive with tolbutamide, the carboxy metabolite is inactive [31]. A minor route of tolbutamide metabolism occurs via butyl chain oxidation at the ω and ω-1 carbons to give primary and secondary alcohol metabolites, respectively, which have minimal antidiabetic activity (Figure 7). An inference can be made from the ratio of the hydroxy metabolites of tolbutamide: when a benzylic methyl group and an alkyl chain are present in the same drug molecule, the preference of metabolic oxidation is for the benzylic methyl group. Substantiation of the inference is given by the higher stability of the benzylic free radical involved in the oxidation of the benzylic methyl group compared to the alkyl-chain free radicals. The stability of the benzylic free radical is due to resonance stabilization [33], as depicted in Figure 8. The metabolic oxidation of the benzylic methyl group occurs as per the mechanism of alkyl hydroxylation shown in Figure 2.

#### 2.2.3. Chlorpropamide

**Figure 9:**
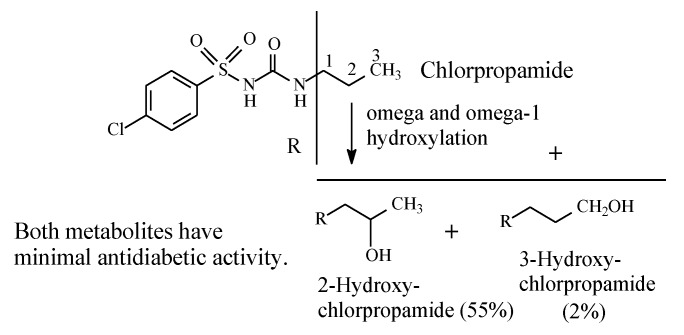
Metabolism of chlorpropamide.

Chlorpropamide (Figure 9) has been developed as a variant of tolbutamide in order to prolong the drug’s antidiabetic effect with the consequent enhancement of potency. Chlorpropamide is slowly metabolized by alkyl-chain oxidation at the ω and ω-1 carbons to give primary and secondary alcohols, respectively (Figure 9) [34]. Both metabolites have minimal antidiabetic activity [20]. It should be noted that in chlorpropamide metabolism, the secondary alcohol (55% of dose) predominates over the primary alcohol (2%) [34] (Figure 9). A possible explanation of this finding resides in the higher stability of the intermediate secondary-propyl free radical compared to the primary-propyl free radical. In the absence of corresponding data on metabolite concentrations, the analogy could be extended to the butyl group in tolbutamide (Section 2.2.2).

#### 2.2.4. Tolazamide

**Figure 10:**
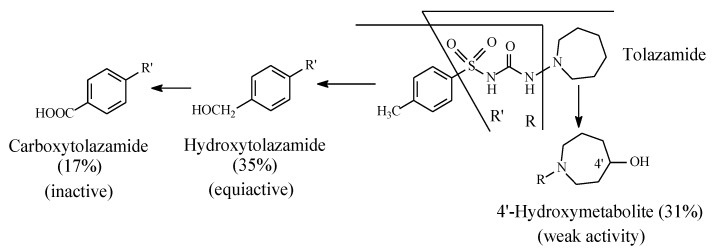
Metabolic pathways of tolazamide.

Tolazamide (Figure 10) contains an azepane ring bonded to the terminal sulfonylurea nitrogen and a methyl group bonded to the aromatic ring. The metabolism of tolazamide is depicted in Figure 12. The azepane ring is oxidized at position 4′ to the 4′ hydroxy group, while the benzylic methyl group is oxidized sequentially to the hydroxymethyl and carboxyl groups [35]. It is noteworthy that the concentrations of the two alcoholic metabolites are almost equal, which leads to the inference that the benzyl and azepanyl free radicals are almost of equal stability. However, as far as activity is concerned, the hydroxymethyl metabolite is equiactive with the parent drug, while the azepanyl alcohol metabolite is only weakly active. Furthermore, the carboxy metabolite resulting from the hydroxymethyl group oxidation is inactive.

#### 2.2.5. Glibenclamide

**Figure 11:**
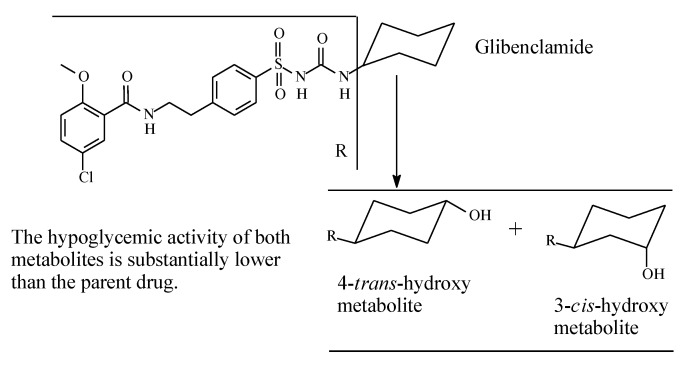
Metabolic pathways of glibenclamide.

Glibenclamide (also known as glyburide) (Figure 11) is a second-generation sulfonylurea oral antidiabetic. It contains a cyclohexyl group bonded to the terminal nitrogen of the sulfonylurea moiety. The cyclohexyl ring forms the main site of metabolism of the drug; it is stereoselectively hydroxylated to 3-*cis* and 4-*trans* isomers (Figure 11) with the latter isomer being the major metabolite [36,37]. The two metabolites have little hypoglycemic effect compared to the parent drug. However, retention of 4-*trans*-hydroxyglyburide may prolong the hypoglycemic effect of the agent in those with severe renal impairment [37].

#### 2.2.6. Glimepiride

**Figure 12:**
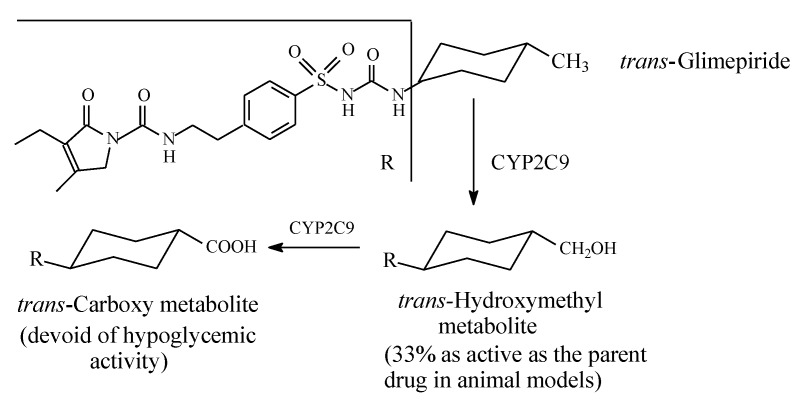
Metabolism of glimepiride.

The cyclohexylmethyl group in glimepiride (Figure 12) allows the drug to exist in *cis* and *trans* isomeric forms; the active antidiabetic form is the *trans* isomer. The latter is metabolized, as shown in Figure 12, through the sequential oxidation of the cyclohexylmethyl group to the hydroxymethyl and carboxy metabolites [38,39]. The hydroxymethyl metabolite is an active antidiabetic in animal models, while the carboxyl metabolite is inactive [39].

#### 2.2.7. Glipizide

**Figure 13:**
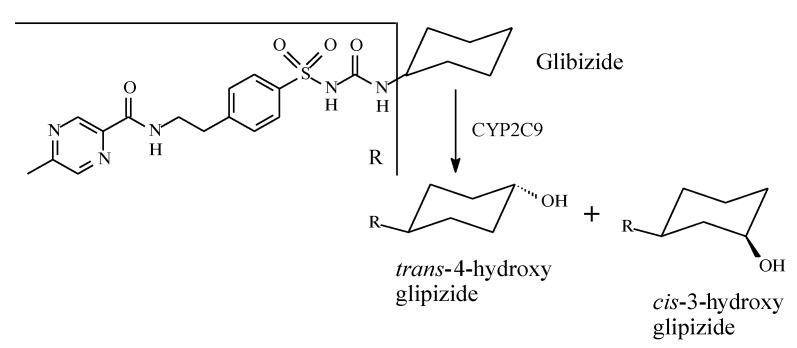
Metabolism of glipizide.

Glipizide (Figure 13) is a second-generation sulfonylurea with R_2_ in Figure 6 as cyclohexyl substituent. It is metabolized by stereoselective hydroxylation to 4-*trans* and 4-*cis*-hydroxyglipizide [40]. No data are available on the activity of the hydroxy metabolites of glipizide.

### 2.3. Barbiturates

Barbiturates are CNS depressants used as sedatives and hypnotics, anesthetics, and anti-seizure drugs. Barbiturates’ primary mechanism of action is inhibition of the central nervous system (CNS). The CNS depression is brought about by stimulating the inhibitory neurotransmitter system in the brain called the gamma-aminobutyric acid (GABA) system. The GABA channel is a chloride channel that has five subunits at its gate. When barbiturates bind to the GABA channel, they cause the chloride ion channel to open, which allows chloride ions into the cells in the brain. The entry of the chloride ions into the brain leads to increased negative charge and alteration of the voltage across the brain cells. This change in voltage makes the brain cells resistant to nerve impulses, thus depressing them [41].

**Figure 14:**
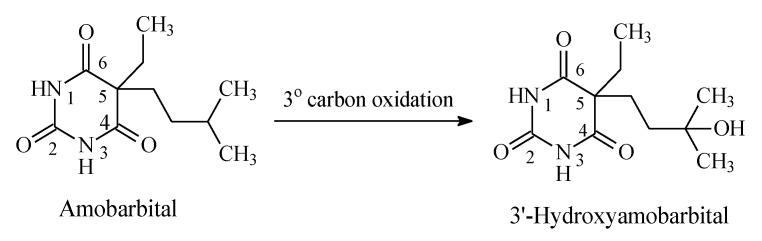
Metabolic pathway of amobarbital.

**Figure 15:**
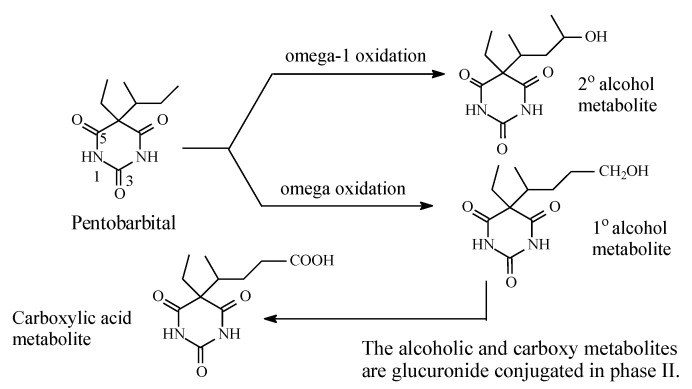
Metabolic pathway of pentobarbital.

Most barbiturates contain alkyl groups of varying lengths. Being lipophilic, these alkyl groups are functionalized by metabolic hydroxylation at different positions. The primary alcohols resulting from oxidation of ω carbons are usually further metabolized to carboxylic acids. Amobarbital and pentobarbital (Figure 14 and Figure 15, respectively) are taken as representative examples of barbiturates that contain alkyl groups. Amobarbital has ethyl and amyl groups bonded to C5 of barbituric acid, while pentobarbital has ethyl and 2-methylbutyl groups bonded to C5 of barbituric acid (Figure 14 and Figure 15). Amobarbital is metabolized by 3′-hydoxylation to give 3′-hydroxyamobarbital as nearly the sole metabolite (Figure 14) [42,43]. On the other hand, the 2-methylbutyl group in pentobarbital undergoes ω and ω-1 metabolic oxidation to give primary- and secondary-alcohol metabolites, respectively (Figure 15) [44]. The primary alcohol metabolite of pentobarbital is further oxidized to the carboxylic acid. The three metabolites of pentobarbital (the two alcoholic metabolites and the carboxyl metabolite) are further conjugated by glucuronic acid in phase II (Figure 15) [44]. The alcoholic and the carboxylic acid metabolites of pentobarbital, and their glucuronide conjugates, are inactive as sedative-hypnotics [44]. In contrast to the alcoholic metabolites of pentobarbital, 3′-hydroxyamobarbital has not been reported to undergo glucuronide conjugation—possibly because, being a tertiary alcohol, it is sterically hindered from such a metabolic pathway. In addition to metabolism, redistribution of barbiturates has been reported to play an important role in their deactivation [45]. Redistribution of the lipophilic barbiturates from the brain to other body compartments, such as adipose tissue, will lead to a reduction of their effective concentration at the receptors in the brain, thus leading to a loss of sedative-hypnotic activity. On the other hand, amobarbital is rapidly metabolized; however, its extended activity has been attributed to the 3′-hydroxy metabolite, which is present in diminished concentration but is, nevertheless, longer acting than the parent drug [43].

### 2.4. Miscellaneous

#### 2.4.1. Valproic Acid

**Figure 16:**
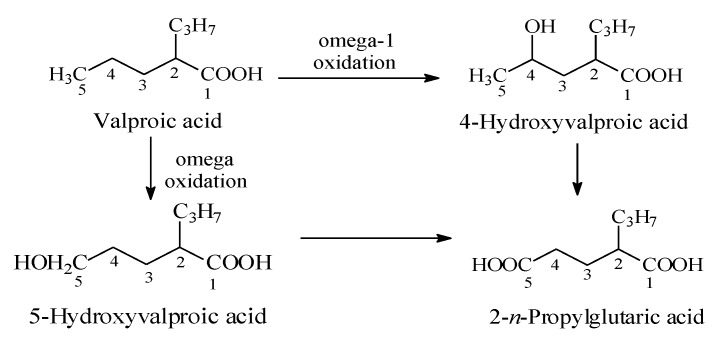
Metabolic pathways of valproic acid.

Valproic acid (Figure 16) is an anticonvulsant drug used in the treatment of epilepsy. Its mechanism of action involves the blockage of voltage-gated sodium channels and increased brain levels of gamma-aminobutyric acid (GABA) [46]. Valproic acid is mainly metabolized by oxidation to alkene and hydroxy products [47,48]. The two major hydroxy metabolites are the 4- and 5-isomers. The primary alcoholic metabolite (i.e., 5-hydroxyvalproic acid) is further oxidized to the carboxylic acid to give 2-*n*-propylglutaric acid (Figure 16). The substantial reduction in anticonvulsant activities of the valproic acid hydroxy and carboxy metabolites has been attributed to their increased molecular size and surface, steric effects, and reduced log P, all of which are features that lower the extent of blood-brain barrier crossing [47,48].

#### 2.4.2. Risperidone/Paliperidone

**Figure 17:**
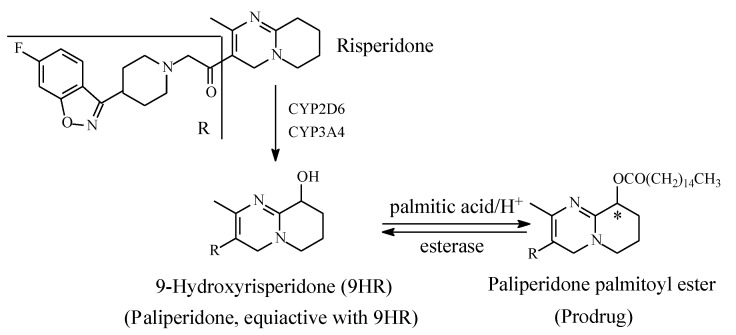
Metabolism and metabolite prodrug development of risperidone into paliperidone palmitate.

Risperidone (Figure 17) blocks the formation of serotonin and dopamine, thus decreasing psychotic and aggressive behavior. By targeting serotonin 5HT2A and D2 receptors, risperidone is considered an atypical antipsychotic drug and is used in the treatment of schizophrenia. In addition, it is used off-label in the treatment of ADHD in children. The metabolism of risperidone is stereoselectively catalyzed (i) by CYP2D6 at the aliphatic heterocycle to give the major enantiomer (+)-9-hydroxyrisperidone, and (ii) by CYP3A4 to (−)-9-hydroxyrisperidone (Figure 17) [49,50,51,52,53]. Both enantiomers are equiactive with risperidone and have been developed into the racemate antipsychotic drug paliperidone [49,50,51,52,53]. Almo and Lopez-Mufioz (2013) [51] have reviewed the clinical use of both risperidone and paliperidone, stressing the pharmacokinetic and pharmacodynamic bases on which the metabolite drug has been developed. Further, the metabolically formed hydroxy group in paliperidone has been esterified with palmitic acid to give paliperidone palmitate. Paliperidone palmitate (Figure 17) is a depo-long-acting injectable prodrug formulation indicated for a single dose to be given once monthly [53]. The active drug is released in the blood by esterase hydrolysis.

Paliperidone palmitate is an example of a prodrug that has been developed from a metabolite drug; hence, it can be described as a metabolite prodrug. Other metabolite prodrugs will be presented and discussed in due course.

#### 2.4.3. Bupropion

**Figure 18:**
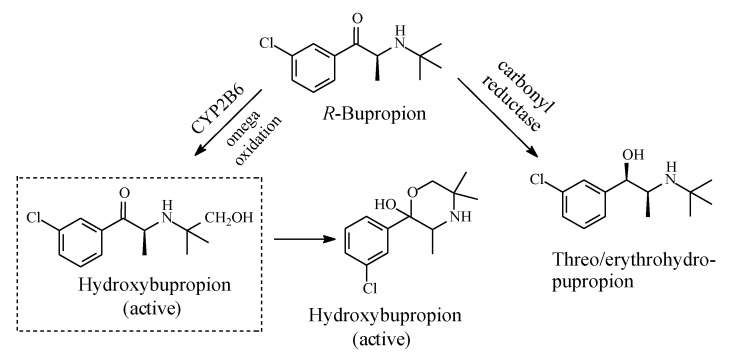
Metabolic pathways of bupropion.

Bupropion (Figure 18) is an atypical antidepressant drug used to treat major depressive disorder (MDD) and seasonal affective disorder; it is also used off-label as a smoking cessation aid. Mechanistically, bupropion enhances both noradrenergic and dopaminergic neurotransmission via reuptake inhibition of the norepinephrine and dopamine transporters. In addition, its mechanism of action may involve the presynaptic release of norepinephrine and dopamine. The major active metabolite of bupropion is hydroxybupropion (Figure 18) [54,55]. The groups in this metabolite are positioned in such a way as to allow for the occurrence of cyclization, thus preventing further oxidation of the hydroxymethyl group to the carboxyl group and the consequent loss of activity. The cyclic metabolite is an active antidepressant [55].

#### 2.4.4. Δ^9^-Tetrahydrocannabinol

**Figure 19:**
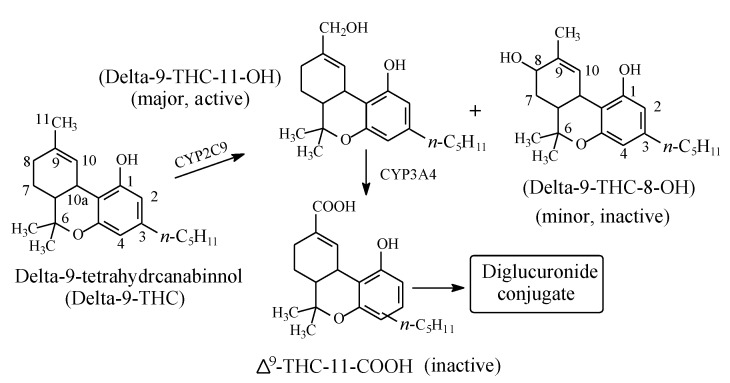
Metabolism of Δ^9^-tetrahydrocannabinol.

**Figure 20:**
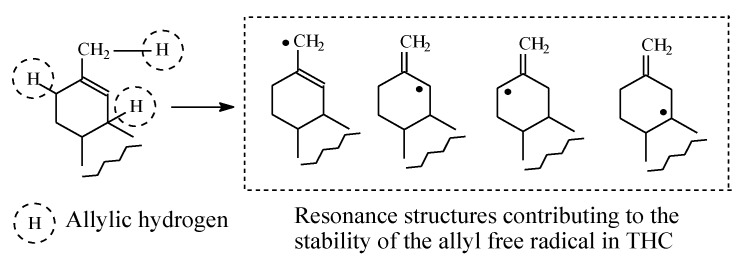
Resonance structures of the allyl free radicals in Δ^9^-THC.

Δ^9^-Tetrahydrocannabinol (Δ^9^-THC, Figure 19) is the psychoactive hallucinogenic constituent in *Cannabis sativa* (hashish and marijuana). It contains three allylic carbons at positions 11, 8, and 10a (Figure 19). The allylic positions at C11and C8 are metabolically hydroxylated, with the former hydroxylation resulting in the major equiactive hydroxymethyl metabolite; due to steric hindrance, position 10a is not hydroxylated. The C11 hydroxymethyl metabolite is further metabolically oxidized to the inactive 11-carboxy-Δ^9^-THC metabolite (Figure 19) [56,57]. The resonance stabilization of the allyl free radical in Δ^9^-THC that accounts for the formation of the major allylic hydroxy metabolite is depicted in Figure 20.

#### 2.4.5. Tolterodine/Fesoterodine

**Figure 21:**
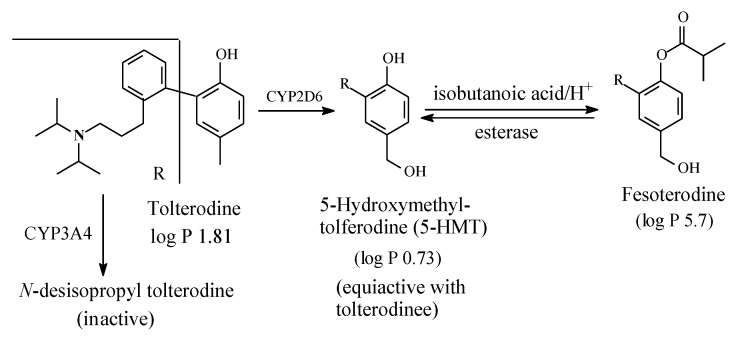
Metabolic pathways of tolterodine and formation of fesoterodine.

Tolterodine (Figure 21) is an antimuscarinic drug used in the treatment of overactive bladder (OAB). As shown in Figure 21, tolterodine is metabolized (i) through mono-deisopropylation to give an inactive metabolite, and (ii) through benzylic-methyl group oxidation to give 5-hydroxymethyl tolterodine (5-HMT), which is equiactive with the parent drug [58,59,60,61]. Despite being equiactive to its parent drug, 5-HMT did not qualify for the status of metabolite drug because of its low log P value of 0.73 and the associated poor bioavailability [58]. However, the problem was resolved by esterifying the aromatic hydroxy (phenolic) group with isobutanoic acid to produce the prodrug fesoterodine, which has a log D_7.4_ value of 5.7 [58] and hence enjoys a substantial improvement in bioavailability.

Fesoterodine is the second example of parent-drug equiactive metabolites to have been developed into metabolite prodrug. The first example from this class of prodrugs is paliperidone palmitate, presented and discussed in Section 2.4.2. Further discussion of metabolite drugs and prodrugs will be given in Section 3.

#### 2.4.6. Terfenadine/Fexofenadine

**Figure 22:**
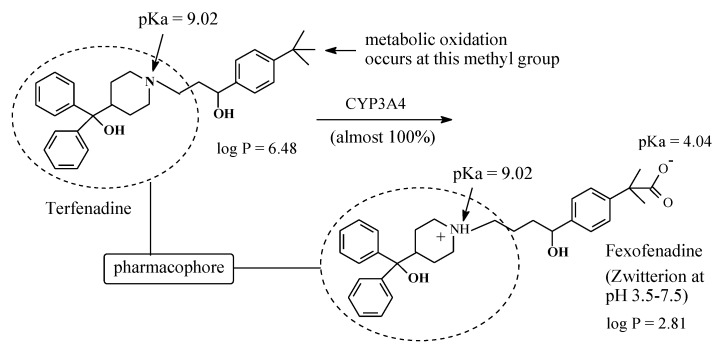
Metabolism of terfenadine to fexofenadine.

Terfenadine (Figure 22) is a second generation H1-antihistamine free of the sedative side effect associated with the first-generation H1-antihistamines. Terfenadine is almost completely metabolized by benzylic-methyl-group oxidation to an equiactive carboxy metabolite, as shown in Figure 22 [62], and it is thus considered a prodrug. However, despite this advantage, terfenadine was withdrawn from clinical use because of its cardiotoxic effect [63]. In the interim, its carboxy metabolite, being free of cardiotoxicity, was developed into a drug of its own right under the name of fexofenadine. As shown in Figure 24, fexofenadine is amphoteric and thus is capable of existing as a zwitterion at physiologic pH [64]. The existence of fexofenadine as zwitterion at physiologic pH may be explained by the carboxylic group’s interaction with the basic pyridinyl nitrogen via folded conformers [65]. Generally, zwitterions do not cross the blood-brain barrier and hence do not cause sedation [65].

#### 2.4.7. Ebastine/Carebastine

**Figure 23:**
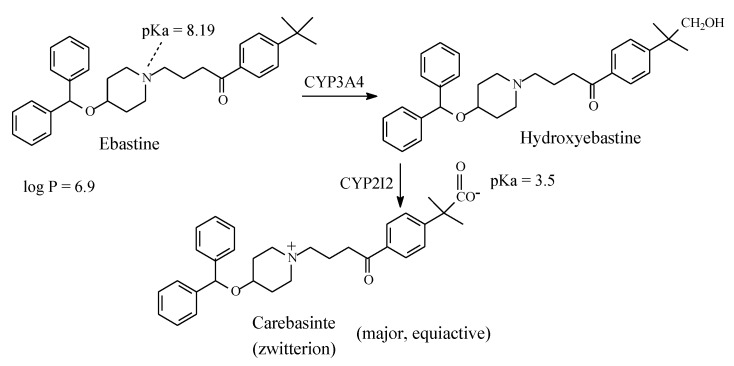
Major pathway of ebastine metabolism.

**Figure 24:**
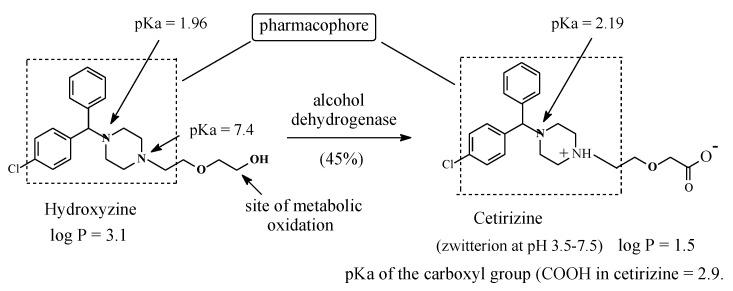
Hydroxyzine metabolism to cetirizine.

Ebastine (Figure 23) is a second-generation non-sedating H1-antihistamine. Its structure is similar to that of terfenadine. As in the latter drug, in ebastine the benzylic methyl group is metabolically oxidized to the carboxyl group after an intermediate step in which hydroxymethyl metabolite is formed as shown in Figure 23. The resulting metabolite, given the name of carebastine, is more active than the parent drug and accounts for nearly all the H1-antihistaminic activity [66]. Despite its high log P value of 6.9 [67], ebastine does not cross the blood-brain barrier, and accordingly it does not cause sedation. On the other hand, carebastine, the active metabolite of ebastine, exists as zwitterion at physiologic pH (Figure 24) and accordingly does not cross the blood-brain barrier. Further, like terfenadine (Section 2.4.6), ebastine is cardiotoxic [68]. It is worth mentioning that, despite carebastine lack of cardiotoxicity relative to its parent drug, it has not been developed into a fully-fledged drug in analogy with fexofenadine (Section 2.4.6). Almirall-Prodesfarma, a Spanish pharmaceutical company, reached stage III in the development of carebastine for the treatment of allergic conjunctivitis and allergic rhinitis, but the company subsequently discontinued the endeavor [69].

### 2.5. Metabolic Conversion of Intrinsic Hydroxymethyl Groups in Parent Drugs to Active Carboxy Metabolites

As has been shown in examples in Section 2.1, Section 2.2, Section 2.3 and Section 2.4, carboxy-metabolites can result from the oxidation of ω-methyl groups in alkyl chains or methyl groups attached to cycloalkyl or aromatic rings. In all the cases cited, the carboxy metabolites were found to be pharmacologically inactive, that is, they did not give the same pharmacological effect as the corresponding parent drugs. However, this observation should not be generalized. Three prominent examples in which intrinsic hydroxymethyl groups are metabolically oxidized to the carboxyl groups with retention of activity are hydroxyzine to cetirizine, salicin to salicylic acid, and losartan to losartan carboxylic acid.

#### 2.5.1. Hydroxyzine/Cetirizine

Hydroxyzine (Figure 24) is a first-generation H1-antihistamine. H1-antihistamines are generally lipophilic in nature, a property that causes them to cross the blood-brain barrier to cause sedation as a main side effect [70]. Hydroxyzine is primarily metabolized by oxidation of the primary alcoholic group to give the equiactive carboxyl metabolite (Figure 24) [71]. Being appreciably more hydrophilic than hydroxyzine and capable of existing as a zwitterion at the physiologic pH of 7.4 (Figure 24), the metabolite does not cross the blood-brain barrier and therefore does not cause sedation [72]. As a result of this pharmacokinetic advantage, the carboxy metabolite of hydroxyzine has been developed into a second-generation H1-antihistamine of its own right under the name of cetirizine [62,63]. The existence of cetirizine as zwitterion may be explained by analogy to fexofenadine in Section 2.4.6. Hydroxyzine and cetirizine are used concurrently in clinical settings; night urticaria may be a suitable indication for the sedative hydroxyzine, while in allergic reactions demanding alertness, cetirizine is indicated [72].

#### 2.5.2. Salicin/Salicylic Acid/Aspirin

**Figure 25:**
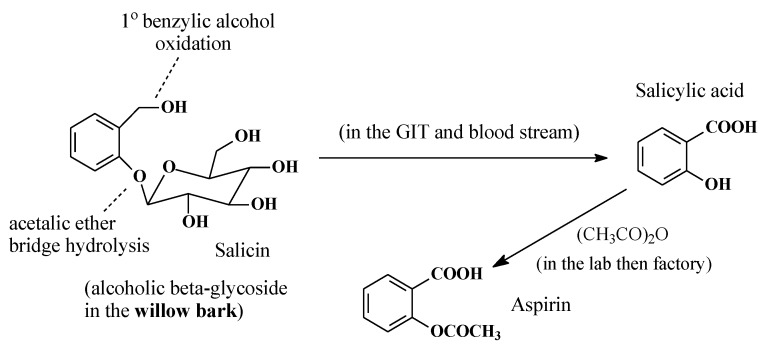
Metabolic pathway of salicin.

Salicin (Figure 25) is a natural product found in the bark of the willow tree. The major turning point for salicylate medicines came in 1763, when a letter from the English chaplain Edward Stone was read at a meeting of the Royal Society. Stone’s letter described the dramatic power of the willow bark extract to cure intermittent fever, pain, and fatigue [73]. As shown in Figure 25, the metabolism of salicin to salicylic acid involves acetalic ether bridge hydrolysis (reminiscent of aromatic-alkoxy dealkylation) to a phenolic group as well as primary alcohol oxidation to the carboxyl group. The latter metabolic pathway is the subject of this section.

Research into willow bark extract culminated in 1899, when the German drug company, Bayer, prepared aspirin by acetylating the phenolic hydroxy group in salicylic acid, which was believed to cause gastric irritation and bleeding [74]. However, subsequent research has proven that the acetyl group in aspirin is crucial to aspirin’s mode of action as a COX inhibitor in the treatment of inflammation. Through transacetylation, aspirin acetylates the alcoholic hydroxy group of the serine moiety in COX, thus inhibiting it from catalyzing prostaglandin biosynthesis [75].

In addition to being one of the most widely used anti-inflammatory, analgesic, and antipyretic drug, aspirin is now renowned for its use as a thrombolytic agent to prevent blood clotting in patients prone to stroke [76,77,78]. Furthermore, its preventive role in colorectal cancer has almost been established [79,80], and it is now being actively researched for other cancers [79,80].

Three factors played significant roles in the design and development of aspirin: (i) nature, by providing salicin from the willow bark; (ii) metabolism, by converting salicin to salicylic acid; and (iii) medicinal chemistry, by blocking the phenolic hydroxy group of salicylic acid by acetylation. Therefore, from a developmental perspective, aspirin can be described as a natural-product-metabolite-synthetic drug, while salicin can be considered a natural prodrug.

#### 2.5.3. Losartan/Losartan Carboxylic Acid

**Figure 26:**
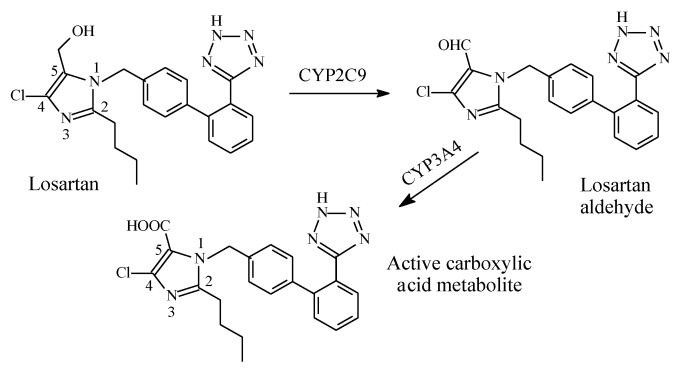
Metabolism of losartan.

Losartan (Figure 26) is a selective, competitive angiotensin II receptor type (AT1) antagonist used as antihypertensive. Through the route shown in Figure 26, the 5-hydroxymethyl group in losartan is metabolized by cytochrome P450 to the 5-carboxylic acid group through intermediate aldehyde formation [81,82,83]. This metabolic route accounts for 14% of losartan dose; the remainder of the drug is excreted unchanged [82,83]. The carboxy metabolite of losartan has 10–40 times the activity of the parent drug [82,83]. Since losartan is only partially converted into an active form, it is not considered a typical prodrug.

According to Foye [81], the hydroxymethyl group in losartan can be replaced by other groups including carboxy, keto, or benzimidazole, to give active ARB drugs. Such groups interact with the AT_1_ receptor via either ionic, ion-dipole, or dipole-dipole bindings. The considerable increase in activity of the carboxy metabolite of losartan compared to the parent drug may be explained by the metabolite’s increased affinity to the receptor caused by the stronger ion-ion or ion-dipole binding due to the ionized carboxylate group at physiologic pH compared to the hydrogen bond binding of the hydroxyl group in the parent drug.

### 2.6. Metabolic Oxidation of Methylene Groups Alpha (α) to Carbonyl and Imino Groups

**Figure 27:**
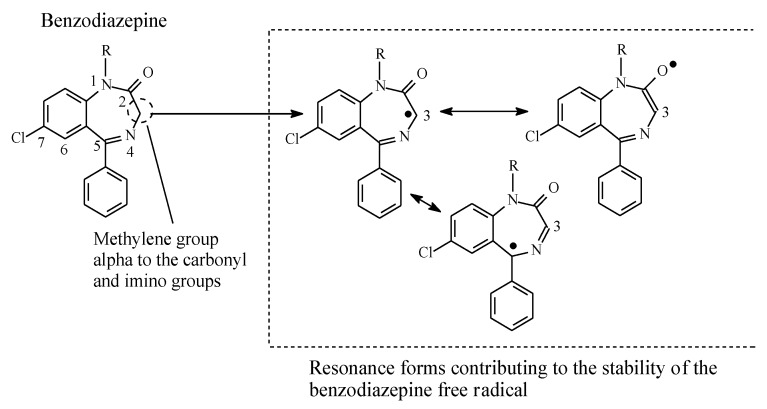
Resonance stabilization of the benzodiazepine free radical.

Generally, methylene groups alpha to carbonyl as well as imino groups undergo metabolic oxidation via mixed function oxidases [84,85]. Examples of drugs in which such groups are found are diazepam and alprazolam within the benzodiazepine class, whose members are used as tranquilizers, hypnotics, or anticonvulsants. The mechanism of metabolic oxidation involves, as a first step, the formation of a resonance-stabilized free radical, as depicted in Figure 27. A hydroxyl group will then be transferred to the free radical in accordance with the mechanism of metabolic alkyl oxidation shown in Figure 2.

#### 2.6.1. Diazepam

**Figure 28:**
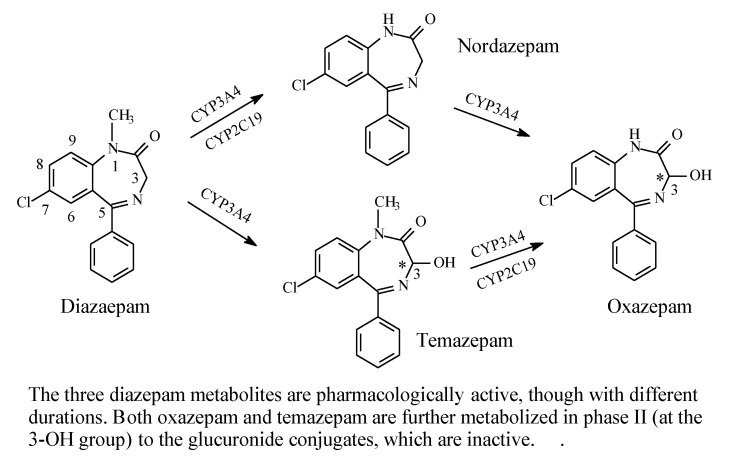
Metabolic pathways of diazepam.

As shown in Figure 28, diazepam is mainly metabolized by hydroxylation at the carbon atom α to the carbonyl and imino groups at position 3, as well as by *N*-dealkylation [86,87,88]. Both metabolic routes give equiactive products with respect to diazepam, though with modified pharmacokinetic properties that affect the drugs’ duration of action. Both hydroxylation at position 3 and *N*-dealkylation result in increased metabolite polarity and hence enhanced metabolite elimination. In addition, glucuronide conjugation taking place at the metabolically generated hydroxy group results in fast elimination and deactivation of the metabolites.

The metabolic hydroxylation of diazepam at position 3 results in the generation of chiral centers in both temazepam and oxazepam (Figure 28). However, despite the presence of several reports in the literature describing the separation of the enantiomers of the drugs [89,90,91,92], studies investigating the activity of their separated enantiomers are lacking.

#### 2.6.2. Alprazolam

**Figure 29:**
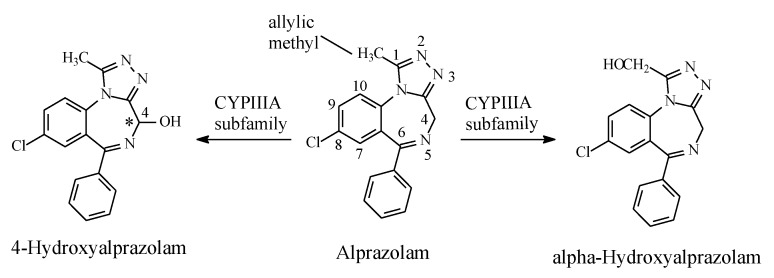
Metabolic pathways of alprazolam.

The triazolobenzodiazepine alprazolam (Figure 29) is metabolized (i) by hepatic microsomal oxidation at C4, which is alpha to two imino moieties, to give 4-hydroxyalprazolam, and (ii) at the methyl group at position 1 to give α-hydroxyalprazolam (Figure 29). Both metabolites have decreased benzodiazepine receptor affinity compared to the parent drug [93].

### 2.7. Alkyl-Moiety Metabolic Hydroxylation in Prodrug Activation

#### 2.7.1. Oxazaphosphorines

**Figure 30:**
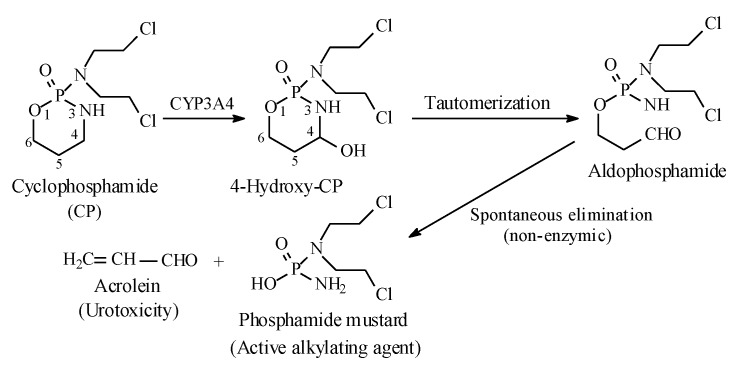
Metabolic activation of cyclophosphamide by the 4-methylene-group hydroxylation.

**Figure 31:**
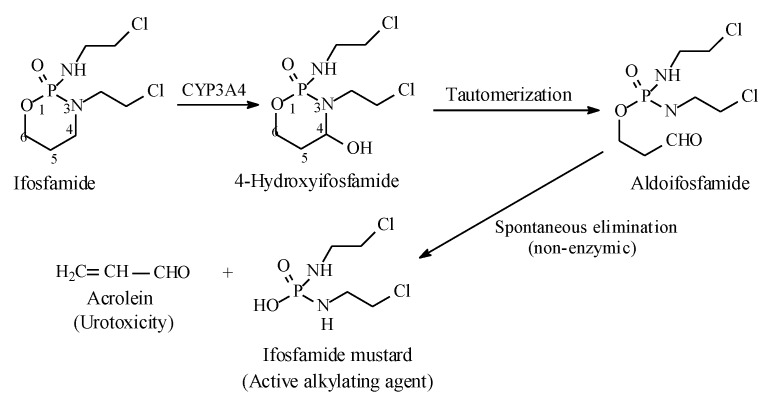
Metabolic activation of ifosfamide by the 4-methylene group hydroxylation.

**Figure 32:**
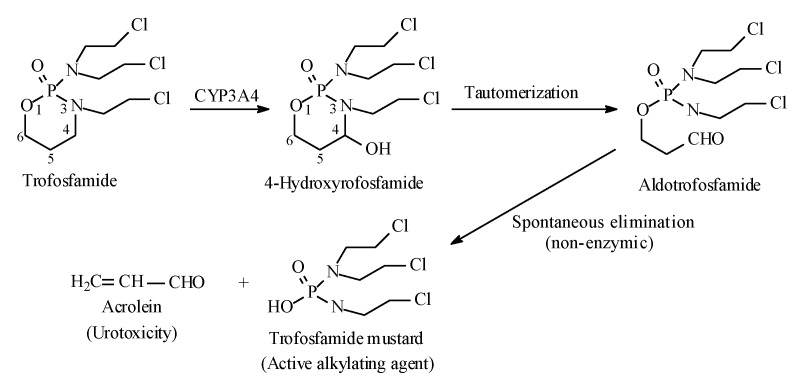
Metabolic activation of profosfamide by the 4-methylene-group hydroxylation.

**Figure 33:**
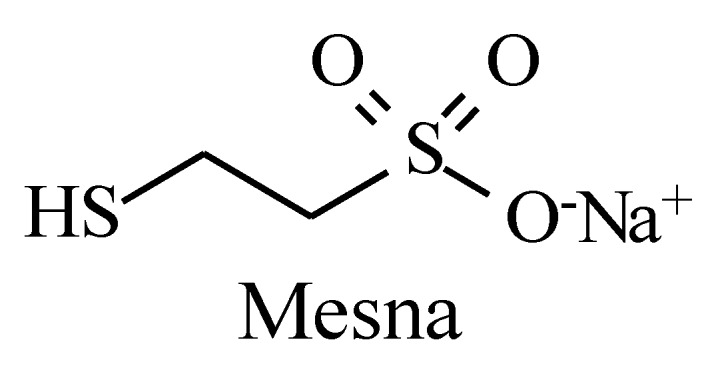
Structure of mesna.

Metabolic alkyl-moiety hydroxylation in prodrug activation is best exemplified by the three oxazaphosphorine alkylating anticancer prodrugs, cyclophosphamide, ifosfamide, and profosfamide [94,95]. The metabolic and chemical processes that lead to the activation of the three drugs in vivo are respectively illustrated in Figure 30, Figure 31 and Figure 32. The first step in the activation process is the metabolic hydroxylation of the 4-methylene group of the common structural feature, the cyclophosphamide. Generally, carbons α to heteroatoms in heterocycles are activated by metabolic oxidation [96]. Next, the secondary alcohol so produced will tautomerize to the aldehydic group to give the aldo tautomer. This is followed by spontaneous non-enzymic elimination of the aldehydic neutral fragment, acrolein, to give the active alkylating agent, the nitrogen mustard. Acrolein causes hemorrhagic cystitis, an adverse effect that can be offset by the concurrent administration of mesna. The mechanism of action of mesna involves the formation of a highly water-soluble conjugate of acrolein that is excreted in the urine [97] (Figure 33).

#### 2.7.2. Aryl-Dialkyl-Triazines

**Figure 34:**
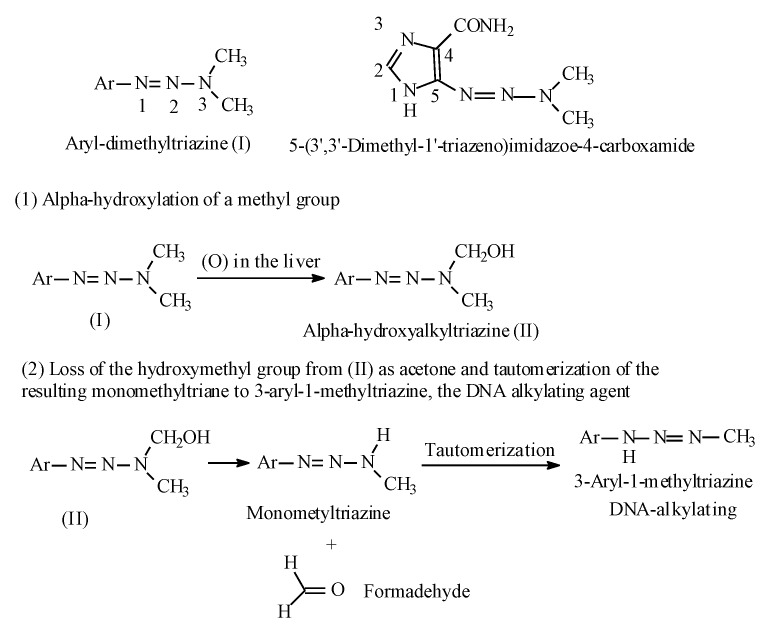
Aryl-dialkyl-triazines and their metabolic activation.

Another class of anticancer prodrugs that are activated by alkyl-group metabolic hydroxylation is the aryl-dialkyl-triazines [98,99]. The antitumor 1-aryl-3,3-dimethyltriazines have the general structure shown in Figure 34. The prototype of this class of drugs is 5-(3,3-Dimethyl-1-triazeno)imidazole-4-carboxamide (DTIC) (Figure 34), used in the treatment of malignant melanoma. The metabolic activation of the aryl-dialkyl-triazines is illustrated in Figure 34.

## 3. Further Interpretations

When studying the effect of drug molecules’ metabolic hydroxy and carboxy functionalization of alkyl groups on metabolite pharmacologic activity, several factors should first be considered. These factors include:

(a) the extent of formation of the hydroxy and carboxy metabolites
(b) the hydrophobicity of the parent drug and hydrophilicity of the metabolites
(c) the drug’s mechanism of action
(d) the favored site of metabolic hydroxylation in drug molecules containing more than one alkyl group
(e) the molecular size increase and steric effect resulting from the replacement of the small hydrogen atom in the alkyl group in the drug molecule by the larger and bulkier hydroxyl or carboxyl group in the metabolite molecule
(f) the creation of new metabolite-receptor binding mechanisms, e.g., hydrogen bonding, ion-pairing, and ion-dipole, in contrast to van der Waals binding of the alkyl groups

Generally, the extent of the metabolic oxidation of carbon atoms in drug molecules’ alkyl chains depends in part on the class of the carbon atom, which in turn dictates the stability of the resulting free radicals: benzylic, allylic > tertiary > secondary > primary > methyl. On the other hand, the hydrophilicity of an alcoholic hydroxyl group is determined by the strength of the intermolecular hydrogen bonds it forms. Due to steric effects, the strength of hydrogen bonding in the different classes of alcohols follows the sequence primary > secondary > tertiary [100]. The order of the hydrophilicity of primary, secondary, and tertiary alcohols follows the same sequence.

From the aliphatic hydroxy and carboxy metabolites of the cases surveyed in this review, we observe three effects on the pharmacologic activity of the metabolites relevant to their parent drugs: loss, attenuation, or retention.

### 3.1. NSAIDS

Loss of pharmacologic activity has been observed for ibuprofen upon metabolic hydroxylation of the isobutyl group at C1, C2, and C3 (Figure 3). In analogy with the *O*-demethylation of the methoxy-group-containing NSAIDS discussed in the first part of this review series [12], both pharmacodynamic and pharmacokinetic effects may account for the ibuprofen isobutyl-hydroxy metabolites’ loss of pharmacologic activity. The SAR of ibuprofen dictates that the branched isobutyl moiety is essential for optimum COX-inhibitory effect; in this context, *n*-butyl substitution has led to significant loss of activity [101]. This finding should imply that each of the methyl groups in the isobutyl moiety occupies a small hydrophobic pocket in COX, enabling a pharmacophoric effect that is essential for optimum activity. The hydroxy and carboxy groups in metabolite III and metabolite IV, respectively (Figure 3), are detrimental to the hydrophobic binding of the isobutyl group, and accordingly, they precipitate a loss of COX-inhibiting activity [102]. Further, by replacing a hydrogen atom in the isobutyl group in ibuprofen, the bulkier hydroxyl or carboxyl groups in the metabolites will impart molecular-size increase and steric effects—factors that are detrimental to optimum binding between isobutyl group and COX [102]. In addition, being hydrophilic, the hydroxyl or carboxyl group will increase the water solubility of the metabolites, hence leading to their elimination and termination of their action. Furthermore, glucuronide conjugation of the hydroxyl and carboxyl groups will considerably enhance the prospects of metabolite elimination and activity termination through substantially increasing aqueous solubility.

### 3.2. Sulfonylurea Oral Antidiabetics

For the sake of discussing the effect of metabolic oxidation of alkyl and aliphatic cyclic groups in the sulfonylurea antidiabetics, we dissect the general structure of these agents, as depicted in Figure 5. To reiterate, in the first-generation sulfonylureas (Section 2.2), R_1_ (Figure 7) is a small lipophilic group, such as methyl or chloro, while R_2_ is an alkyl or aliphatic cyclic group. According to Foye (2020) [26], the R_1_ groups do little to increase the binding efficiency of the pharmacophore to the ATP-sensitive K^+^ channel. As such, R_1_ groups may be playing weak auxiliary pharmacophoric and/or auxophoric roles. On the other hand, the R_2_ groups in both first- and second-generation sulfonylurea antidiabetics have the auxophoric role of optimizing the pKa of the sulfonylurea group to ~5. At this pKa value, a sulfonylurea anion is formed that is essential for interaction with the pancreatic β-cell subtypes (SUR1, SURA1, and SUR2A) through ion-ion and ion-dipole bindings [103]. Generally, metabolic change at auxiliary pharmacophores or auxophores is associated with retention of pharmacologic activity [12]. However, a discrepancy is observed for some members of the first-generation sulfonylurea antidiabetics in this respect. For instance, while the aliphatic-ring hydroxy metabolite of tolazamide (Figure 12) is active with a prolonged duration of action, the counterpart metabolite of acetohexamide (Figure 8) is inactive.

The lipophilic methyl group at R_1_ in the general structure of sulfonylureas (Figure 5) is metabolized by oxidation, via hydroxymethyl formation, to the carboxyl group with loss of activity in both tolbutamide and tolazamide (Figure 7 and Figure 9, respectively). Generally, the loss of activity caused by metabolically formed carboxyl groups can be explained by two effects. Firstly, the carboxyl group is almost fully ionized at the physiologic pH of 7.4. Secondly, the carboxyl group is, in most cases, glucuronide conjugated in phase II. These two effects will result in a substantial increase of water solubility and elimination of the metabolite with the consequent loss of activity due to reduced effective concentration of the metabolite at the receptor. It is noteworthy that the metabolic functionalization of the lipophilic benzylic methyl group to the carboxyl group in tolbutamide (Figure 7), with the consequent enhanced elimination and loss of activity, has led to the development of chlorpropamide (Figure 9). By employing bioisosterism, medicinal chemists replaced the benzylic methyl group in tolbutamide with a chloro group, which is not prone to metabolism, to obtain chlorpropamide. Due to this manipulation of metabolic stability, chlorpropamide can be used at a lower dose and frequency than tolbutamide [104].

In the second-generation sulfonylurea antidiabetics, the small lipophilic groups of the first generation at R_1_ (Figure 5) have been replaced by the larger *p*-(β-arylcarboxyamidoethyl) group, such as in glimepiride (Figure 14), in order to attain strong binding affinity to the ATP-sensitive K^+^ channel [26]. Metabolism of this group is not within the scope of this review.

### 3.3. Barbiturates

The metabolic oxidative hydroxylation of alkyl chains in barbiturates has resulted in variable levels of activity subject to the class of the resulting alcohol. In pentobarbital (Figure 17), the primary and secondary alcohols, respectively resulting from ω and ω-1 oxidation, are sufficiently hydrophilic to jeopardize the hydrophobicity requirement for blood-brain barrier crossing [105]. In addition to hydrophilicity, the factors of increased steric effect, molecular size, and surface area may come into play to hinder the hydroxy metabolite from fitting in the receptor, thus leading to either attenuation or loss of activity as governed by the extent of each factor. On the other hand, in amobarbital (Figure 16), metabolic oxidation occurs mainly at the ω-1 tertiary carbon, resulting in a tertiary alcohol, 3′-hydroxyamobarbital. Despite being less active, 3′-hydroxyamobarbital has been reported to be responsible for the sedative-hypnotic activity of amobarbital [43]. With reduced hydrogen bonding ability, and the consequent diminishment of hydrophilicity, due to steric effects in tertiary alcohols, 3′-hydroxyamobarbital is expected to cross the blood-brain barrier in sufficient concentration to produce sedative-hypnotic effects.

The loss of activity of the carboxy metabolites of barbiturates may be explained similarly to the NSAIDS-carboxy metabolites (Section 3.1).

### 3.4. Accounting for the Activity of the H1-Antihistamines’ Carboxy Metabolites: Hydroxyzine, Terfenadine, and Ebastine

Methyl groups that are bonded to aromatic or cycloalkyl rings, or terminal in alkyl chains (i.e., ω methyls) in drug molecules are usually oxidized to inactive carboxy metabolites through the formation of mostly active hydroxymethyl intermediates. The loss of pharmacologic activity indicates that the methyl groups in such cases play pharmacophoric roles, at least of an auxiliary nature. However, when the methyl or hydroxymethyl group is distant from a predetermined pharmacophore, the situation is different: metabolic oxidation of either group to the carboxyl group does not cause loss of activity of the resulting metabolite. This has been the case with the three H1-antihistamines hydroxyzine, terfenadine, and ebastine, which are respectively metabolized to equiactive cetirizine (Figure 24), fexofenadine (Figure 22), and carebastine (Figure 23).

Hydroxyzine is a first generation H1-antihistamine. With a log P value of 3.5 [106], it is hydrophobic enough to cross the blood-brain barrier, interact with cholinergic, serotonergic, and adrenergic receptors and cause sedation [107]. On the other hand, cetirizine, the carboxy-metabolite drug of hydroxyzine, has a log P value of 1.5 [108] and exists as zwitterion at physiologic pH of 7.4. Due to these properties, cetirizine does not cross the blood-brain barrier and does not accordingly cause sedation. As shown in Figure 24, in both hydroxyzine and cetirizine, the metabolically exchanged groups are distant from the pharmacophore, and accordingly, the two drugs are therapeutically equiactive as H1-antihistamines. The ethoxyethanol group in hydroxyzine and the ethoxyacetic acid group in cetirizine each play an auxophoric role. A similar situation can be observed for the terfenadine/fexofenadine H1-antihistamine pair (Figure 23). However, here, the carboxyl group in fexofenadine plays a pharmacodynamic rather that a pharmacokinetic role. Terfenadine causes heart arrhythmias by blocking the hERG channel K^+^ current [109]. On the other hand, the ionized carboxylate group (COO^−^) in fexofenadine reduces this blockage by over three orders of magnitude, thus rendering this drug almost free of the cardiotoxic effect [110].

The inference that can be made from the two H1-antihistamine pairs presented above is that when metabolic changes occur at groups distant from the primary pharmacophores (i.e., at auxophoric groups), the original pharmacologic activity will not be affected. Further, beneficial pharmacokinetic and/or pharmacodynamic modifications may result in the metabolites warranting their development into fully-fledged drugs. An extended definition of pharmacophores is given in Section 3.6.7.

### 3.5. Aspirin Is an NSAID of Its Own Disposition

In salicin (Figure 27), the acetalic group is metabolically converted to a hydroxyl group in a reaction reminiscent of *O*-dealkylation, while the hydroxymethyl group is oxidized to the carboxyl group to give salicylic acid. The phenolic hydroxyl group in salicylic acid was suspected to be the cause of stomach irritation and bleeding, and it was hence esterified by acetic anhydride to give aspirin. However, later, it was proven [111,112,113] that the gastrointestinal adverse effects of aspirin were associated with the inhibition of COX1 and accordingly the inhibition of PGE1 formation, i.e., synthesis of the prostaglandin involved in the protection of gastric mucosa against acid attack. Sometime then elapsed before the mechanism of the anti-inflammatory activity of aspirin was understood to be caused by acetylation of the serine moiety in COX [114]. That being the case, the benzene ring and the carboxyl group in aspirin are likely playing auxiliary pharmacophoric roles by properly anchoring the aspirin molecule in the COX-active cavity, thus facilitating the transfer of the acetyl group to the serine moiety.

### 3.6. Subtexts Arising from Hydroxy and Carboxy Metabolic Functionalization of Alkyl Moieties in Drug Molecules

The aim of this section is to provide focused information on some general and specific issues that have been extracted from the individual cases of alkyl-moiety metabolic hydroxy and carboxy functionalization. The information presented and discussed includes definitions, significance, implications, and/or applications of selected topics, which include:

3.6.1. Metabolism of methyl groups in drug molecules
3.6.2. Metabolic hydroxylation of alicycles and aliphatic heterocycles in drug molecules
3.6.3. Inferences from hydroxymethyl group in drug metabolites regarding origin and significance
3.6.4. Development of metabolite drugs and prodrugs from metabolites equiactive with parent drugs
3.6.5. Pharmacologic activity of carboxy metabolites
3.6.6. Significance of the carboxy metabolite of Δ^9^-tetrahydrocannabinol
3.6.7. Primary and auxiliary pharmacophoric properties

#### 3.6.1. Metabolism of Methyl Groups in Drug Molecules

Methyl groups assume their importance in drug molecules due to their small size, higher steric effect with respect to the hydrogen atom, hydrophobicity, isosterism with a number of groups, and historical inclusion in drug molecules. They are found in drug molecules at ω-carbons in both straight- and branched-chain alkyls, as substituents in aromatic (benzene) rings and alicycles, and as substituents in secondary and tertiary amino moieties. In branched-chain alkyls, methyl groups are found as isopropyl, isobutyl, or *tert*-butyl moieties. In all of these forms, the methyl group is metabolically oxidized by CYP450 enzymes to the hydroxymethyl group. The sequential oxidation of the latter group to the carboxylic acid follows in most cases via primary alcohol formation. When there is more than one equivalent methyl group in a drug molecule, only one group will be metabolically oxidized.

#### 3.6.2. Metabolic Hydroxylation of Alicycles and Aliphatic Heterocycles in Drug Molecules

Six-membered alicycle (cyclohexyl) and heterocycle (piperidinyl) groups are often encountered in drug molecules of various pharmacologic classes. For the most part, rings are stereoselectively metabolized by hydroxylation at the positions 3 and 4, which are less sterically hindered, compared to other positions (in the ring), to form *cis* and *trans* isomers. Pharmacologic action may also be a function of stereoselective metabolism. For instance, the oral antidiabetic acetohexamide is mainly metabolized to *trans*-4′-hydroxyacetohexamide, which is inactive. The cyclohexyl ring in glibenclamide is metabolically oxidized to 3-*cis* and 4-*trans-*hydroxy metabolites with a substantial attenuation of antidiabetic activity. A similar effect has been observed for tolazamide, in which the azepane ring is metabolically hydroxylated at position 4 (Figure 11) with a substantial loss of activity.

**Figure 35:**
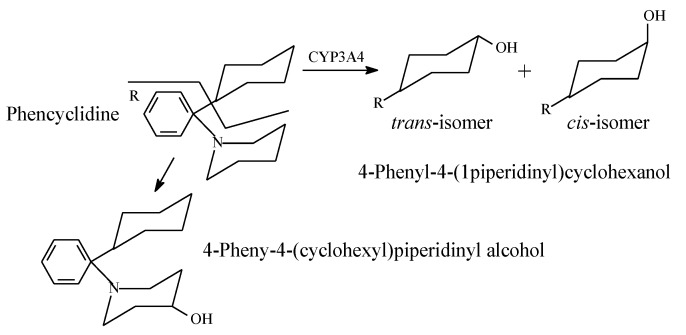
Metabolic pathways of phencyclidine.

An interesting case of metabolic hydroxylation of alicycles and aliphatic heterocycles is given by the psychotropic drug phencyclidine, which contains both cyclohexyl and piperidinyl groups. Phencyclidine is mainly metabolized by hydroxylation at position 4 of the cyclohexyl ring to the active *cis*- and *trans*-4-phenyl-4-(1-piperidinyl)cyclohexanol (Figure 35) [115]. In addition, the piperidinyl ring in phencyclidine is metabolically hydroxylated to a minor extent at position 4 to give 4-phenyl-4-(1-coclohexyl)piperidinyl alcohol [116]. The pharmacologic activity of the piperidinyl-hydroxy metabolite of phencyclidine has not been reported. A tentative inference can be made heeding the phencyclidine metabolic hydroxylation example: when an alicycle and aliphatic heterocycle are parts of the same molecule, metabolic hydroxylation favors the alicycle over the heterocycle.

#### 3.6.3. Inferences from Hydroxymethyl Metabolites

Hydroxymethyl groups (-CH_2_OH), either intrinsic to drug molecules or metabolically formed, play pharmacophoric and/or auxophoric roles. Here, we highlight the significance of the hydroxymethyl group as derived from the relevant cases in Section 2.

Hydroxymethyl groups may be intrinsic to drug molecules, or they may result from the metabolic oxidation of methyl groups bonded to aromatic or alicyclic rings or terminal methyl groups in alkyl chains—i.e., ω carbons. Intrinsic or metabolically formed hydroxymethyl groups are almost invariably metabolically oxidized to carboxyl groups.

The hydroxymethyl metabolites are almost invariably equiactive with the parent drugs, whereas the carboxy metabolites (resulting from the sequential oxidation of the hydroxymethyl metabolites) are mostly inactive with only a few exceptions being equiactive with the parent drugs. These exceptional cases are those in which the hydroxymethyl groups are distant from the primary pharmacophore.

The fact that hydroxymethyl metabolites invariably retain the pharmacologic activity due to the parent drug probably reflects the auxiliary pharmacophoric status of the methyl group from which they have resulted.

Of the hydroxymethyl metabolites that are equiactive with their parent drugs, only that of tolterodine has been developed into a prodrug, which carries the name of fesoterodine (Figure 20).

#### 3.6.4. Development of Metabolite Drugs and Prodrugs from Parent-Drug Equiactive Metabolites

The metabolite drugs presented in Section 1 include the H1-antihistamines cetirizine (Figure 24) and fexofenadine (Figure 22), the carboxy metabolites of hydroxyzine and terfenadine, respectively. Both drugs are more hydrophilic than their respective parent drugs and are capable of existing as zwitterions at physiologic pH. Due to these properties, the two drugs do not cross the blood-brain barrier, and accordingly, they do not cause sedation. In addition, the carboxyl group in fexofenadine seems to offer an ionic binding site that is responsible for the removal of cardiotoxic adverse effects from the parent drug terfenadine. The advantages of both cetirizine and fexofenadine that warranted their development into metabolite drugs may be described as intrinsic. However, cases are known in which these advantages are artificially produced, such as in metabolite prodrugs. In such cases, the metabolite is equiactive with the parent drug, but due to high hydrophilicity, it suffers the disadvantages of low bioavailability and short duration of action. Chemists have responded to this situation by producing ester prodrugs. The two metabolite prodrugs presented in Section 2 are the antipsychotic paliperidone palmitate (Section 2.4.2, Figure 17) and the antimuscarinic fesoterodine (Section 2.4.5, Figure 21). The development of ester prodrugs of hydroxy metabolites equiactive with their parent drugs to attain improved pharmacokinetic properties may be extended to other cases if sufficiently warranted.

#### 3.6.5. Pharmacologic Activity of Carboxy Metabolites

In most of the surveyed drugs in Section 2, where terminal methyls in alkyl chains (ω-carbons), benzylic methyls, and methyls directly bonded to alicycles are metabolically oxidized to the carboxy group, a loss of pharmacologic activity due to the parent drug has been observed. However, two cases, terfenadine and ebastine, are unique in that the oxidation of the benzylic methyl group has resulted in retention of pharmacologic activity. In these two particular cases, the metabolic oxidation of the methyl group to the carboxyl group has taken place at positions distant from the primary pharmacophores: terfenadine and ebastine (Figure 20 and Figure 21, respectively). Of all the metabolically generated polar functional groups in drug molecules, the carboxyl group stands alone in that it is almost completely ionized at the physiologic pH of 7.4. If such metabolic change occurs at a pharmacophoric site, the new state of ion-pairing interactions replacing the van der Waals interaction of the methyl group will tend to change the pharmacodynamics of the parent drug, leading to a loss of activity. On the other hand, the ionized carboxyl group introduces a pharmacokinetic dimension—which substantially enhances the polarity, water solubility, and elimination of the metabolite as per se or as the glucuronide conjugate, thus causing a significant reduction of the metabolite’s effective concentration at the receptor. It is noteworthy that when a carboxyl group is involved in zwitterion formation with an aliphatic amino group, such as in fexofenadine, carebastine, and cetirizine, it (the carboxy group) will not be subject to glucuronide conjugation. In fact, glucuronide conjugation has not been reported as a metabolic route for any of the three aforementioned drugs.

#### 3.6.6. Significance of the Carboxy Metabolite of Δ^9^-Tetrahydrocannabinol

Δ^9^-Tetrahydrocannabinol (Figure 20) is the major psychoactive constituent of *Cannabis sativa*. The use of *Cannabis* products, hashish and marijuana, is illegal in many countries and is punishable by law. The detection of cannabis-product use is based on urinalysis of the constituent metabolites. For this purpose, presumptive immunoassays have been developed based on the carboxy metabolite of Δ^9^-tetrahydrocannabinol, i.e., carboxy-Δ^9^-tetrahydrocannabinol (Figure 21). Confirmation tests of the presence of this metabolite are carried out by chromatography-mass spectrometry methods such as GC-MS after trimethylsilyl derivatization or LC-MS [117,118].

#### 3.6.7. Primary and Auxiliary Pharmacophores

In the first part of this review series [12], we classified the pharmacophore as primary and auxiliary (secondary or logistic) based on the “message/address” concept suggested by Dr. Portoghese [119]. The case of fexofenadine development as a metabolite drug of terfenadine (Figure 24) due to the cardiotoxicity of the latter has prompted us to extend the definition of an auxiliary pharmacophore. An auxiliary pharmacophore is a group that may play one of two roles: (a) properly anchoring the primary pharmacophore in the active site of the receptor or enzyme, or (b) interacting with a site other than the primary site (i.e., an auxiliary site) to produce or override an adverse effect or to account for an off-label use of the drug. Hence, the cardiotoxic effect of terfenadine may be explained by the interaction of the three benzylic methyl groups with an auxiliary receptor via van der Waals binding. On the other hand, in fexofenadine, one of the benzylic methyl groups (in terfenadine) has been metabolically oxidized to a carboxyl group, which interacts with the auxiliary site via ionic binding. Due to its higher strength, ionic binding strongly predominates over van der Waals binding and thus dictates, in part, the nature of the pharmacologic activity of drugs in which it occurs.

## 4. Conclusions

The occurrence and extent of alkyl moiety hydroxy functionalization in drug molecules is predictable based on the feasibility of intermediate free radicals’ formation and stability. On the other hand, the pharmacologic activities of the alkyl moieties-hydroxy and carboxy metabolites may be predicted based on analogy with the reviewed cases. All hydroxymethyl metabolites are pharmacologically equiactive with their parent drugs while most carboxy metabolites are inactive. The development of metabolite ester prodrugs has an extendable potential when the equiactive hydroxy metabolite is characterized by poor bioavailability and/or short duration of action. The pharmacologic activities of the hydroxy and carboxy metabolites resulting respectively from alkyl or hydroxymethyl moiety functionalization are explicable on pharmacodynamic and/or pharmacokinetic grounds. In some cases, metabolic hydroxy and carboxy functionalization of alkyl or hydroxymethyl moieties has enabled distinctions to be made between primary pharmacophores, auxiliary pharmacophores, and auxophores.

## Figures and Tables

**Table 1:** Moieties metabolized by hydroxy and carboxy functionalization.

Aliphatic Moieties Metabolized by Oxidative Hydroxylation	Metabolic Products
Alkyl (linear or branched)	Primary, secondary, or tertiary alcohols, depending on the class of substrate carbon; primary alcohols are further oxidized to carboxylic acids
Unhindered methylene groups in alicycles and aliphatic heterocycles (usually at the farthest position from the monosubstituent)	Secondary alcohols
Benzylic methyl groups	Primary alcohols, followed by oxidation to carboxyl group
Methyl groups bonded to alicycles or heterocycles	Primary alcohols, followed by oxidation to carboxyl groups
Methylene groups alpha to both carbonyl and imino groups	Secondary alcohols
Carbons α to a heteroatom in a heterocycle	Secondary alcohol; usually followed by carbonyl compound (aldehyde or ketone) elimination
Allylic carbons	Primary alcohols in open-chain alkyls and secondary alcohols in alicycles
